# Image-guided confirmation of a precision pull-through procedure during laparoscopically assisted anorectoplasty in an open MRI operating theater: first application in an infantile case with anorectal malformation

**DOI:** 10.1186/s40792-021-01298-1

**Published:** 2021-09-20

**Authors:** Shun Onishi, Chihiro Kedoin, Masakazu Murakami, Nayuta Higa, Akihiro Yoshida, Kazutoshi Onitsuka, Takahiro Moriyama, Koji Yoshimoto, Satoshi Ieiri

**Affiliations:** 1 Department of Pediatric Surgery, Research Field in Medical and Health Sciences, Medical and Dental Area, Research and Education Assembly, Kagoshima University, 8-35-1, Sakuragaoka, Kagoshima City, 890-8520 Japan; 2 Department of Neurosurgery, Graduate School of Medical and Dental Sciences, Kagoshima University, Kagoshima, Japan; 3 Department of Anesthesiology and Critical Care Medicine, Graduate School of Medical and Dental Sciences, Kagoshima University, Kagoshima, Japan

**Keywords:** Anorectal malformation, Laparoscopically assisted anorectoplasty, Recto-bulbar urethral fistula, Image-guided surgery, Magnetic resonance imaging, Open MRI, Operating theater

## Abstract

**Background:**

Image-guided surgery with an open magnetic resonance imaging (MRI) system is applied for brain tumors in the neurosurgery field, but has rarely been reported in pediatric surgery. We report our initial experience of intraoperative confirmation of precision rectal pull-through during laparoscopically assisted anorectoplasty (LAARP) in an open MRI operating theater for pediatric patients with anorectal malformation (ARM).

**Case presentation:**

A 3.0 kg term male neonate was delivered with anorectal malformation. An invertogram revealed the intermediate type. Transverse colostomy was made on the left upper abdomen. The recto-bulbar urethral fistula (RBUF) was diagnosed by a distal colostogram and voiding cystourethrogram. LAARP was planned at 6 months of age. Because this was the first procedure in which the pediatric abdomen had been scanned in an open MRI operating theater in our institution, we scanned his pelvic floor under sedation 3 weeks before the operation using the open MRI system in our operation room. We performed the operation with 4 trocars. The peritoneal reflection was carefully incised and the rectum was dissected. The RBUF was resected. The center of the muscle complex was detected at the perineal skin with an electrical nerve stimulator, and a 7-mm longitudinal skin incision was made on the perineal lesion for anoplasty. The muscle complex and the pubo-rectal sling were confirmed laparoscopically using a 3.5-mm bipolar forceps connected to the electrical nerve stimulator. Anoplasty was performed between the rectal stump and perineal skin. After anoplasty, the patient was scanned with open MRI under general anesthesia. We attached the quadrature-detection (QD) head coil around the patient’s pelvis and inserted him in the gantry. A 0.45-T open MRI clearly revealed that the pulled through rectum was located in the center of the muscle complex on T2-weighted images. The postoperative course was uneventful. Oral intake was started on post-operative day 1. Postoperative dynamic urography showed no complication (e.g., leakage or residual fistula).

**Conclusions:**

We successfully performed LAARP for ARM, with intraoperative confirmation of precision rectal pull-through in an open MRI operating theater. Further cases are required to evaluate the application of open MRI systems in pediatric surgery.

## Background

Surgical management for anorectal malformation (ARM) has changed over time. Posterior sagittal anorectoplasty (PSARP) is a standard surgical management for high type or intermediate type of anal atresia [1]. Laparoscopically assisted anorectoplasty (LAARP) is currently performed worldwide [2, 3]. Regardless of the procedure used for pull-through of the rectum, the aim of the definitive operation is to pull the rectal pouch through the center of the muscle complex. Conventionally, pediatric surgeons have used electrical nerve stimulation to detect and confirm the center of the external sphincter; however, other objective and repeatable evaluation methods that could be performed intraoperatively were not reported.

Image-guided surgery with an open magnetic resonance imaging (MRI) system is the frequently performed in the neurosurgery field [4]. In the pediatric surgery field, cases involving the resection of undifferentiated sarcoma and pelvic rhabdomyosarcoma using an open MRI navigation system have been reported [5, 6]. Because MRI is especially advantageous for soft-tissue mass visualization, we planned to use MRI to detect the center of the muscle complex during LAARP.

We herein report our initial experience of the intraoperative confirmation of precision rectal pull-through during LAARP for an infant patient with ARM in open MRI operating theater.

## Case presentation

**Fig. 1:**
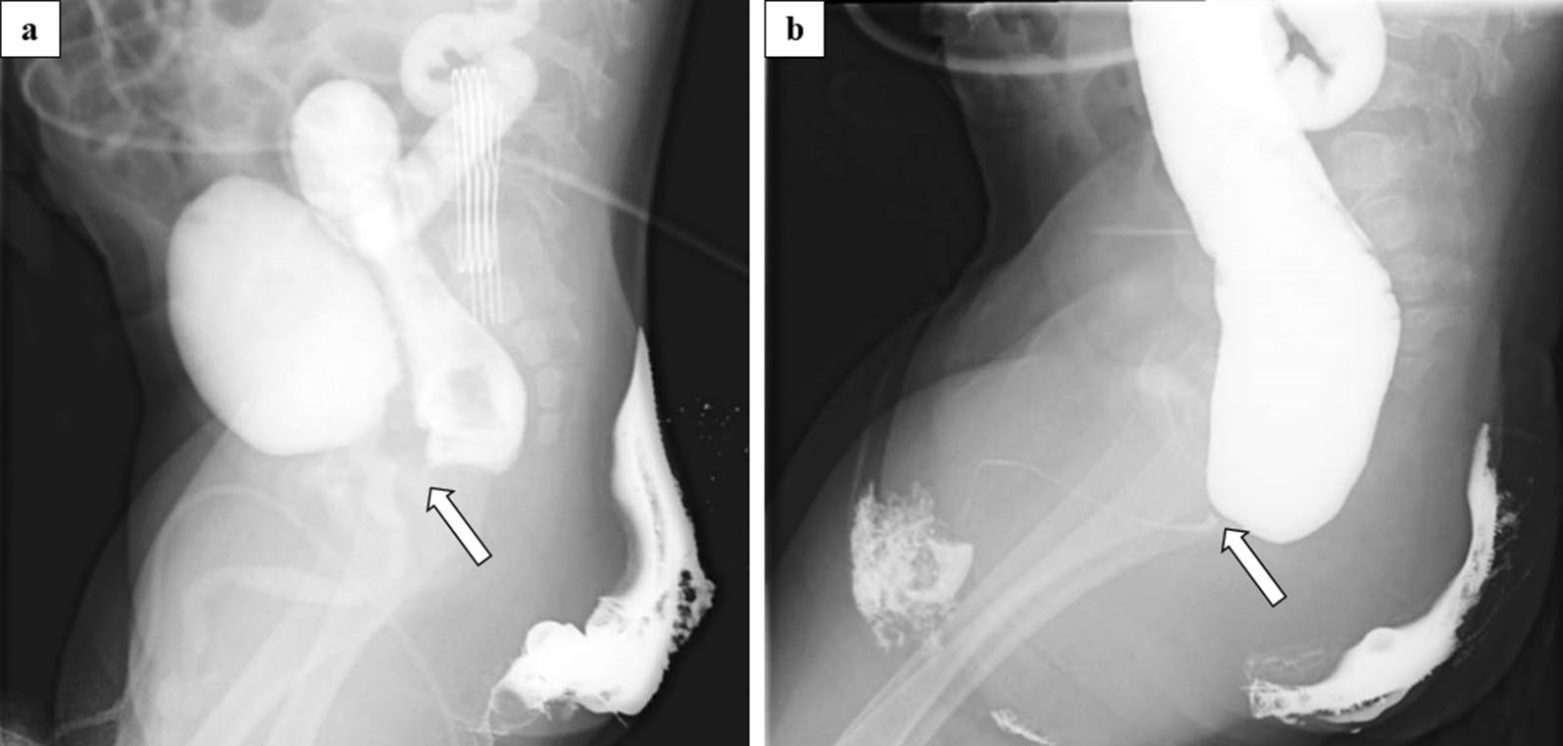
**a** Voiding cystourethrogram and distal colostogram. Recto-urethral fistula was suspected (arrow), but was not clearly visualized. **b** High-pressure distal colostgram. Recto-bulbar urethral fistula (RBUF) was detected (arrow)

A 3.0 kg term male neonate was delivered with an anorectal malformation. An invertogram revealed the intermediate type. Transverse colostomy was made at the left upper abdomen. The recto-bulbar urethral fistula (RBUF) was diagnosed by a distal colostogram (Fig. 1a) and voiding cystourethrogram (Fig. 1b). LAARP with an open MRI scan was planned at 6 months of age.

**Fig. 2:**
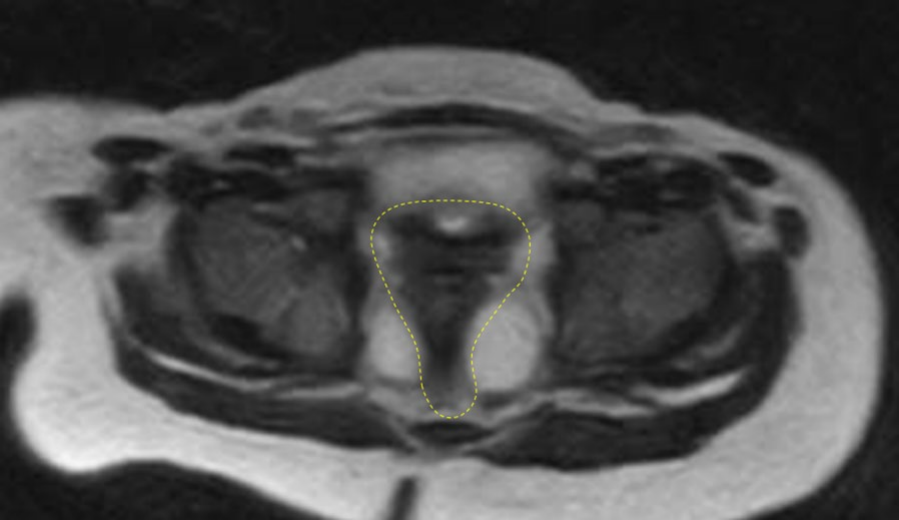
Preoperative MRI scans. T2-weighted images (axial view). Muscle complex are outlined by dot line

Because this was the first case involving the scanning of an infant abdomen with open MRI in our institution, we scanned his pelvic floor under sedation 3 weeks before the operation using an open MRI system (0.45T, APERTO Lucent Plus, Hitachi, Ltd, Japan) in our operation theater. The obtained images showed the muscle complex in pelvis and we presumed that the rectal pull-through procedure would achieve favorable results (Fig. 2).

**Fig. 3:**
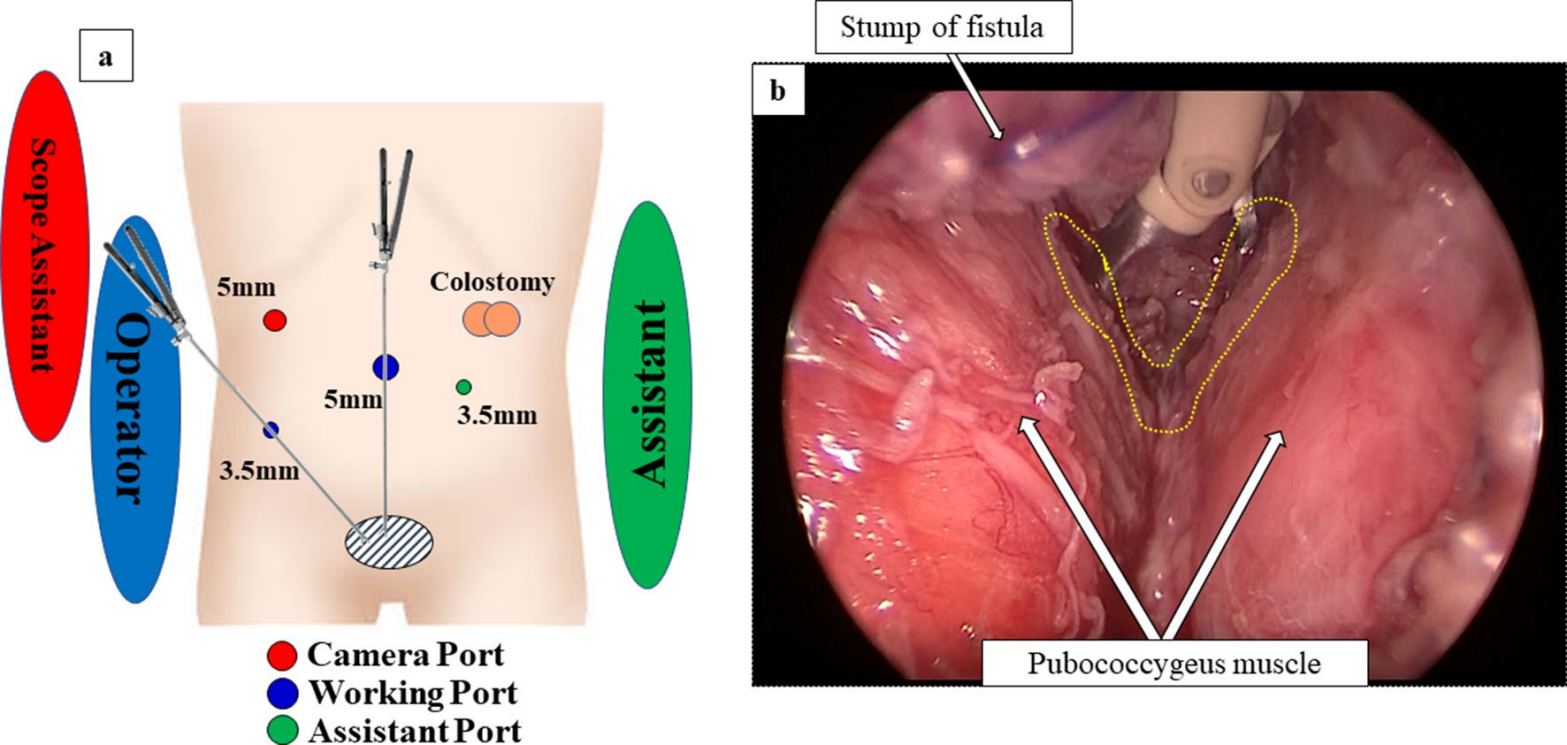
**a** Trocar layout for LAARP. **b** Laparoscopic view of deep pelvic space. After transection of RBUF, muscle complex including pubo-rectal sling (dot line) and pubococcygeus muscle (arrows) were clearly confirmed

Under general anesthesia with tracheal intubation, a 5-mm trocar was inserted at the longitudinal incision of the umbilicus by an open procedure and pneumoperitoneum was established with insufflation of 8 mm Hg CO_2_ (4  L/min). Additional trocars were inserted under the view of a 5-mm laparoscope: a 5-mm trocar at the right upper abdomen for the 30° rigid scope, a 3.5-mm trocar at the right lower abdomen for the operator’s right forceps, and a 3.5-mm trocar at the left side of umbilicus for the assistant’s forceps (Fig. 3a). A 30° 5-mm scope could clearly visualize the small and deep pelvic cavity. To obtain a sufficient operative view and working space, bladder suspension was performed. The peritoneal reflection was carefully incised and the rectum was dissected using 3.5-mm bipolar scissors (RoBi^®^; KARLSTOZ SE & Co. KG, Tuttlingen, Germany). To avoid damaging the pelvic nerve, rectal dissection was performed, with preservation of the layer between the pre-hypogastric nerve fascia and the fascia propria of the rectum. The RBUF was closed by trans-fixing suture using 4–0 absorbable monofilament (PDS Plus, Johnson & Johnson, New Jersey, USA) and then transected with a stapler (Signia™ Small Diameter Reload, Medtronic, Dublin, Ireland) in advance to secure the surgical field and to prevent peritoneal contamination. After additional dissection, the remnant of RBUF was ligated with an ENDOLOOP^®^ Ligature (Johnson & Johnson, New Jersey, USA) and resected just below the urethra with the staples. The center of the muscle complex was detected at the perineal skin with the electrical nerve stimulator, and a 7-mm longitudinal skin incision was made on the perineal lesion for anoplasty. The muscle complex and pubo-rectal sling were also confirmed laparoscopically using a 3.5-mm bipolar forceps connected to the electrical nerve stimulator (Fig. 3b). A Pean forceps was inserted to keep the center of the muscle complex away from the perineal skin incision under a laparoscopic view, and a 5-mm trocar was replaced through the center of the muscle complex. The stapled rectum was pulled through, and anastomosis was performed between the stump and perineal skin for anoplasty.

**Fig. 4:**
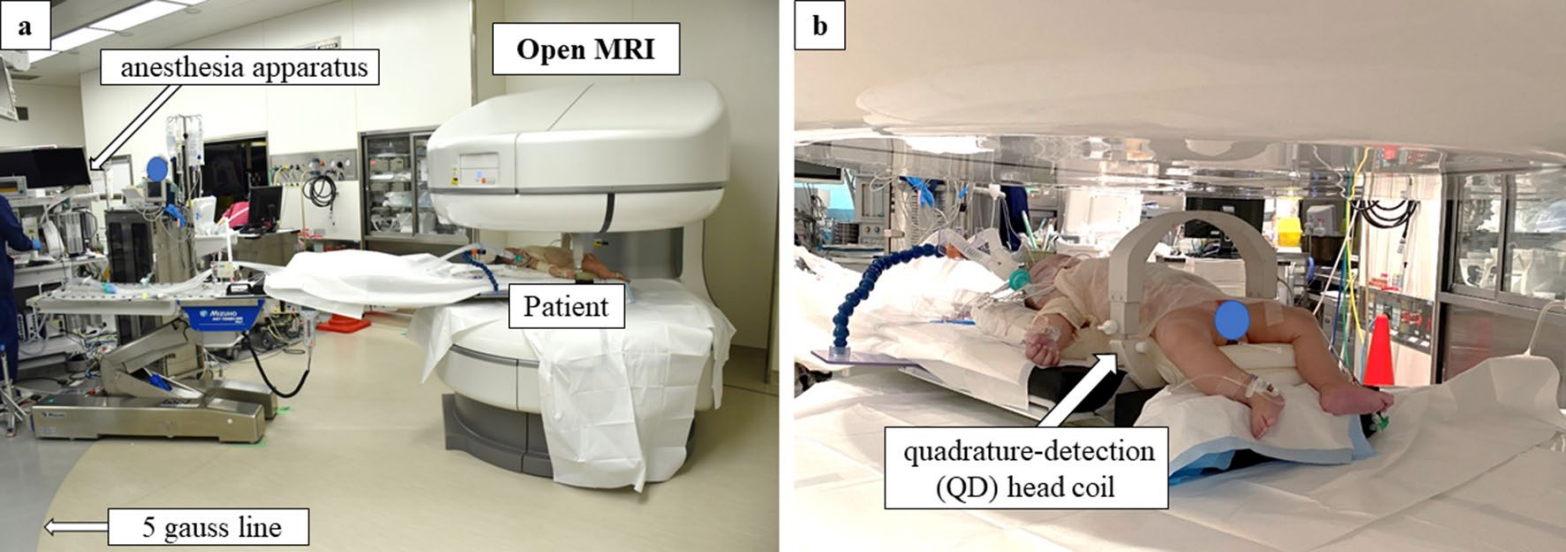
**a** Long respiratory tube between the anesthetic instrument and the gantry was needed for general anesthesia. **b** Patient was inserted into the gantry for open MRI scanning. The quadrature-detection (QD) head coil was attached around the patient’s pelvis

**Fig. 5:**
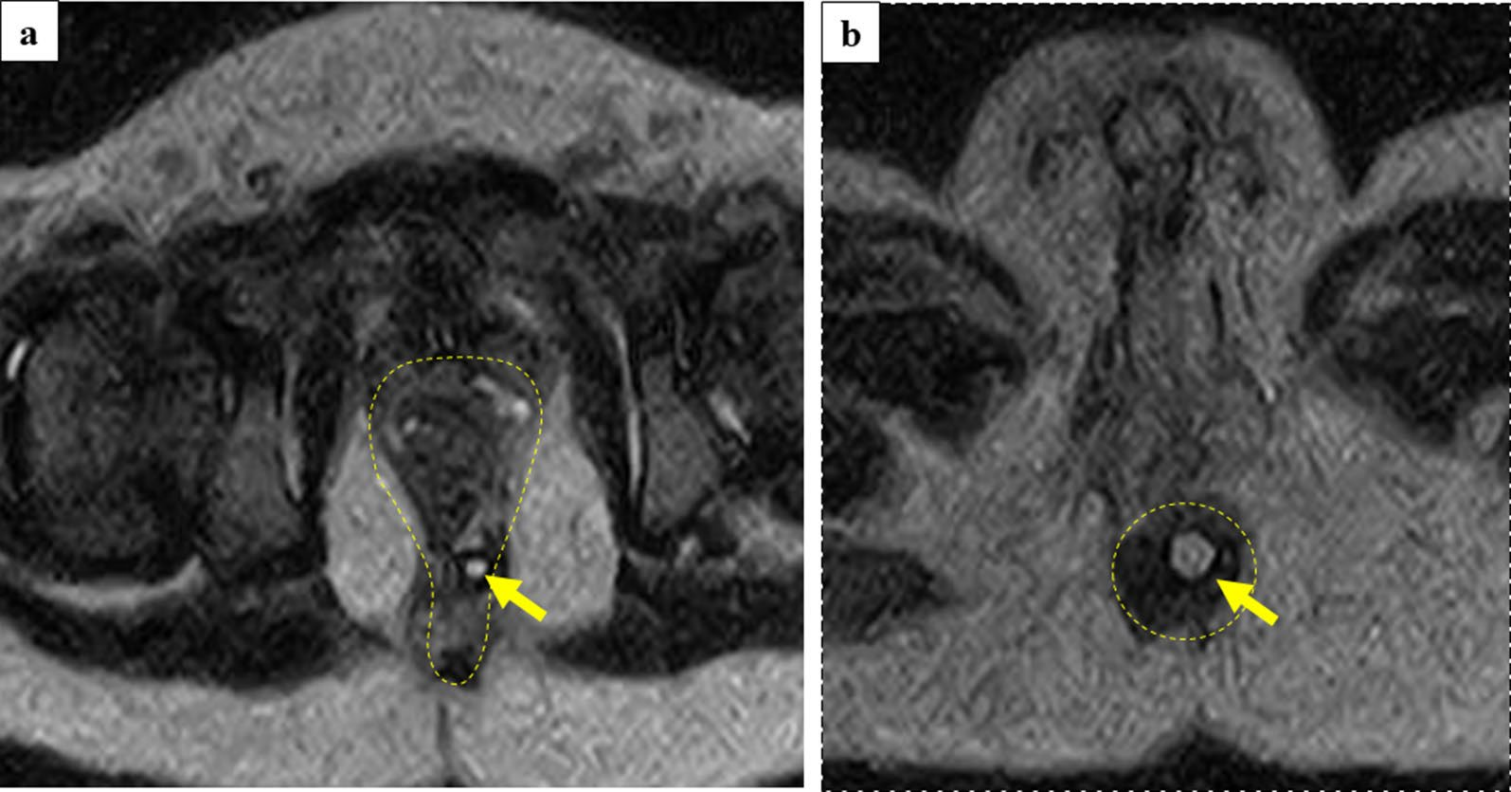
T2-weighted images (axial view). **a** A 0.45-T open MRI scan clearly revealed the rectum in the center of muscle complex (outlined by dot line). To clarify rectal pull-through, a 1 ml syringe filled with physiological saline was inserted in the anus (arrow). **b** Dot line showed the external sphincter muscle. Pull through rectum (1 ml syringe filled with physiological saline: arrow) was confirmed at the center of the external sphincter muscle

After anoplasty, the patient was immediately scanned by open MRI under general anesthesia. We attached the quadrature-detection (QD) head coil around the patient’s body and pelvis and inserted him into the gantry (Fig. 4). To clarify rectal pull-through, 1 ml syringe filled with normal saline was inserted in the neo anus. The neurosurgeon and the radiological engineers scanned the patient. The 0.45-T open MRI scan clearly revealed that the pulled through rectum was located at the center of muscle complex on T2-weighted images (Fig. 5). In addition no residual fistula was recognized. The operation time was 420 min and the scanning time was 40 min.

The postoperative course was uneventful. Oral intake was started on post-operative day 1. Postoperative dynamic urography showed no complications on post-operative 14. He was discharged post-operative day 15. We are planning to perform colonogram and take down the transverse colostomy after 3 months.

## Discussion

The present report was the first application case of LAARP using intraoperative open MRI system for a pediatric patient with ARM. We successfully performed LAARP for ARM, with intraoperative confirmation of the precision of the rectal pull-through procedure. The essential goal of LAARP for ARM is the precise placement of the rectum through the muscle complex and external sphincters, which prevents division and injury of these muscles, which play an important role in the postoperative anorectal function [2]. Inaccurate pull-through would lead to a poor bowel function, including soiling and fecal incontinence. Thus far, confirmation of the pull-through procedure was performed in the postoperative period [7]. If the pulled-through rectum is found to be located in an inappropriate position after the operation, revision is difficult due to adhesion and perirectal scarring, and is also associated with a risk of injury of the pelvic nerve and muscle complex [7]. However, this open MRI operating theater allows pediatric surgeons to evaluate the quality of an operation intraoperatively.

MRI operating theater systems have some advantages over other imaging modalities. First, MRI provides multiple images without an increased risk of radiation exposure to the patient [6]. A low magnetic field does not any affect the human body, even in small infants. Second, regarding the above advantage, repeatable scanning during a short period is feasible. Third, MRI is superior to CT in terms of imaging of the soft tissue, including muscles. Above all of these advantages, in an operating theater equipped with open MRI, preoperative, intraoperative and immediate postoperative scanning are all possible for the real-time evaluation of an operative procedure, including the quality and results. In the neurosurgery field, prior to the introduction of intraoperative imaging modalities, the evaluation of the completion of brain tumor resection depended on the surgeon’s subjective impression. The same traditional decision and subjective evaluation processes were recognized in anorectoplasty for AMR. However, LAARP using a high-resolution image can allow us to confirm and grossly observe the muscle complex, as was demonstrated in this case. The combination of an open MRI operating theater and LAARP could provide an absolutely objective evaluation of the pull-through procedure for ARM. If MRI revealed inaccurate pull-through during the operation, revision surgery could be immediately performed to achieve for accurate pull-through.

Open MRI have some advantage compared with conventional “closed” MRI. Thomas et al. [8] reported the guiding technique for placing the neorectum through the entire sphincter complex with the conventional MRI in operating theater. They moved patients to the operation room from the MRI room after scanning. Open MRI can be established in same room because of lower magnetic field than conventional MRI. We could perform LAARP and scan with open MRI in the same room. In addition, conventional MRI require expensive cost for installation because of reinforcing the operation room.

In this case, we successfully performed accurate pull-through without residual fistula on the first attempt in an open MRI operating theater. The precise placement of the rectum would potentially facilitate the acquisition of a satisfactory postoperative bowel function; however, long-term follow-up is required to evaluate the outcome of the operative procedure. Bjørsum-Meyer et al. [7] mentioned that the presence and thickness of interposed fat between the sphincter complex and the bowel was positively correlated to the postoperative continence score with statistically significance at late follow-up of PSARP operation. We will follow the patient and evaluate his bowel function over the long term to validate the open MRI findings.

An open MRI operating theater is associated with some disadvantages and problems. An MRI scan generally takes longer to perform in comparison to computed tomography (CT) scan or radiography; however, it took approximately 3 min for each open MRI scan in the present case. It is a feasible and useful intraoperative modality for pediatric patients. In addition to the characteristics of MRI systems, we had several technical problems that should be solved before the operation. This is the first case in which the abdomen of a child was scanned with the QD head coil; thus, we scanned the patient using an open MRI system in the operation room to adjust the scanning condition of the pediatric abdomen on the operation day. To set the system, radiology engineers and the neurosurgeon performed several scans to acquire proficiency in open MRI scanning. On the operation day, we scanned the patient under general anesthesia. In our institution, all neurosurgery patients were adults and were scanned during awake craniotomy. A long respiratory tube between the anesthetic instrument and the gantry was needed for general anesthesia (Fig. 3). The dead space of the long tube might cause failure of effective mechanical ventilation because of the small body size of the patient. The anesthesiologist prepared a flow-inflating bag that could be used in the gantry. Fortunately, the anesthetic instruments provided good ventilation during scanning.

Despite the above stated disadvantages and problems, an open MRI operating theater offers promising possibilities for pediatric surgery. We are planning real-time navigation for ARM as a next challenge. Intraoperative MR cholangiopancreatography for choledochal cyst and MR urography for urinary tract disease may be other good indications for the use of an open MRI operating theater. Real-time evaluation would improve the operative quality and postoperative functional outcome in pediatric patients.

## Conclusion

We successfully performed LAARP for ARM, confirming precise rectal pull-through using an open MRI operating theater. Further cases are required to evaluate the usefulness of the application of an open MRI system in the pediatric surgery field.

## Data Availability

The data used in this report are available from the corresponding author on request.

## Abbreviations

LAARP: Laparoscopically assisted anorectoplasty
PSARP: Posterior sagittal anorectoplasty
ARM: Anorectal malformation
MRI: Magnetic resonance imaging
QD: Quadrature detection
CT: Computed tomography
